# Phosphate-Solubilizing Bacteria with Contrasting Arsenate Tolerance Sustain Phosphorus Nutrition and Restrict Arsenic Translocation in Peanut

**DOI:** 10.3390/plants15213332

**Published:** 2026-10-28

**Authors:** Liliana Ludueña, María Soledad Anzuay, Ana Furlan, Natalia Rodríguez, Juan Manuel Peralta, Tania Taurian, Eliana Bianucci

**Affiliations:** 1 Instituto de Investigaciones Agrobiotecnológicas—Consejo Nacional de Investigaciones Científicas y Técnicas (INIAB-CONICET), Departamento de Ciencias Naturales, Facultad de Ciencias Exactas, Físico—Químicas y Naturales, Universidad Nacional de Río Cuarto (UNRC), Ruta 36, Km 601, Rio Cuarto X5800, Córdoba, Argentina; lluduena@exa.unrc.edu.ar (L.L.); manzuay@exa.unrc.edu.ar (M.S.A.); afurlan@exa.unrc.edu.ar (A.F.); ttaurian@exa.unrc.edu.ar (T.T.); 2 Institut National des Sciences Appliquées de Lyon (INSA-Lyon), Université Claude Bernard Lyon 1, Université de Lyon, F-69621 Villeurbanne, France; 3 Departamento de Biología Animal, Biología Vegetal y Ecología (BABVE), Universitat Autònoma de Barcelona (UAB), Campus de Bellaterra, 08193 Cerdanyola del Vallès, Barcelona, Spain

**Keywords:** peanut–microbe interaction, phosphorus nutrition, rhizobial coinoculation, redox response, nodulation, phytoprotection

## Abstract

Arsenate [As(V)] competes with phosphate for uptake, creating a challenge for peanut production in P-limited, As-affected agricultural systems. We investigated whether phosphate-solubilizing plant growth-promoting bacteria (PS-PGPB) with contrasting As(V)-tolerance phenotypes remained functional and benefited peanut when coinoculated with *Bradyrhizobium* sp. SEMIA6144. We screened four PS strains: native *Enterobacter* sp. J49, *Serratia* sp. S119, and *Serratia* sp. J260; and commercial *Pseudomonas pergaminensis* PMT1. J49 and PMT1 tolerated millimolar As(V), whereas S119 and J260 were inhibited at micromolar concentrations. As(V) increased soluble P in J49 and PMT1, maintained it in S119, and decreased it in J260. In J49 and S119, As(V) increased arsenate reductase and superoxide dismutase activities, but only S119 showed lipid peroxidation and induced glutathione-related enzymes, revealing different redox responses under strain-specific exposure conditions. Despite this divergence, both strains converged in planta. At a regionally relevant 3 µM As(V), coinoculation with either strain increased shoot P concentration in tricalcium phosphate (PTC)-supplied plants relative to PTC alone and restricted shoot As to below the method detection limit without significantly reducing root As concentrations. Nevertheless, As accumulated in nodules with J49 but remained undetected with S119. These findings identify integrated strain–plant–rhizobium performance as a key criterion for selecting inoculants that couple P acquisition with restriction of detectable total As translocation to shoots.

## 1. Introduction

Arsenic is a highly toxic, non-essential metalloid whose occurrence in soils and groundwater represents both an agronomic and a public-health concern. Human exposure may occur directly through the consumption of contaminated groundwater and indirectly through food crops that incorporate As from their growing environment, while the metalloid can also impair plant growth and crop productivity [1]. This problem is particularly relevant in southern Córdoba Province, where the unconfined aquifer contains naturally occurring, geogenic As whose mobilization is favoured by alkaline conditions and desorption from oxide and hydroxide surfaces [2,3,4]. Groundwater As concentrations in southern Córdoba range from 8 to 2400 µg/L (approximately 0.1–32 µM), with an average of 463 µg/L (approximately 6.2 µM) and pronounced spatial variation even over distances shorter than 2.5 km [2]. The highest values occur particularly in depressed areas and groundwater-flow transit zones, where alkaline conditions and prolonged water–sediment interaction favour As release, predominantly as As(V) [2]. Seasonal rises in the shallow groundwater table and flooding events may bring As-containing groundwater into the rooting zone, providing a direct pathway for root exposure independently of irrigation. Its regional significance is reinforced by the importance of peanut production: approximately 406,000 ha were planted with peanut in Argentina during the 2025/26 growing season, with Córdoba accounting for approximately 75% of national production and nearly all domestic processing capacity. Moreover, Argentina is among the world’s leading peanut exporters [5].

The effects of arsenate [As(V)] on plant nutrition are closely linked to phosphorus (P) availability. As a chemical analogue of phosphate, arsenate can enter plants and microorganisms through phosphate transport systems, directly linking P acquisition to metalloid exposure and toxicity [6,7]. This interaction is especially relevant in agricultural soils where total P may be abundant but its plant-available fraction remains limited because of precipitation or adsorption processes [8]. Soils of the peanut-producing region of Córdoba are characterized by low P availability, making access to sparingly available P pools an important determinant of crop performance [9,10]. Phosphate-solubilizing bacteria (PSB) can release phosphate from poorly available mineral forms and improve P acquisition by peanut plants [11,12]. By increasing phosphate availability, these bacteria could sustain plant P nutrition under As(V) exposure while modifying the competition between phosphate and arsenate for shared transport systems. Therefore, candidate strains should be evaluated for both their survival under metalloid exposure and their capacity to retain plant growth-promoting functions.

Phosphate-solubilizing plant growth-promoting bacteria (PS-PGPB) can mobilize inorganic P and complement the contribution of symbiotic rhizobia to legume growth and nutrition. In peanut, bacterial mobilization of poorly available P sources has improved P acquisition, biomass production, nodulation, and yield under both controlled and field conditions [10,11,12,13,14]. However, survival under metalloid exposure does not necessarily ensure preservation of these functions. Arsenic can modify bacterial viability and individual PGP traits in a strongly strain-dependent manner, and highly tolerant isolates do not invariably express the functional attributes required for plant growth promotion [15,16,17]. Tolerance must therefore be considered as an integrated physiological phenotype involving not only survival, but also P mobilization, management of oxidative stress, and maintenance of functions relevant to the host plant.

Previous work using 3 µM As(V) demonstrated that even this low concentration induced significant physiological and redox alterations in nodulated peanut plants and that the *Bradyrhizobium* partner strongly influenced plant performance and organ-specific As partitioning [18]. These findings provided the biological basis for using 3 µM As(V) in the present study and for evaluating whether native peanut-associated PS-PGPB from the same agricultural region could complement the performance of *Bradyrhizobium* sp. SEMIA6144. Evidence that PS-PGPB can improve peanut performance under As exposure remains limited [19], particularly when these bacteria interact with a defined N-fixing symbiont. However, shoot N content declined under As(V) with SEMIA6144 but was maintained with C-145, revealing a trade-off between restricting As translocation and sustaining plant N nutrition. Coinoculation with non-rhizobial, nodule-associated PS-PGPB could provide a complementary strategy to improve peanut growth and nutrient acquisition while maintaining limited As movement towards aerial tissues.

We therefore hypothesized that peanut-associated PS-PGPB with contrasting As(V)-tolerance phenotypes would retain functional P-solubilizing capacity and, when coinoculated with *Bradyrhizobium* sp. SEMIA6144, sustain peanut P nutrition and restrict As translocation under an environmentally relevant As(V) exposure.

## 2. Results

### 2.1. Effect of As(V) on Bacterial Growth and Viability

All four strains remained viable throughout the 12-h exposure period, although viable counts were differentially affected by increasing As(V) concentrations (Supplementary Figure S1; Supplementary Table S1). Preliminary screening showed that S119 and J260 failed to grow at the millimolar concentrations tested; therefore, their exposure range was narrowed to the micromolar level. Viable counts increased over time in all four strains, but S119 and J260 showed marked inhibition within the micromolar range, whereas PMT1 and J49 maintained growth at As(V) concentrations up to 4 mM. Based on these contrasting responses, S119 and J260 were classified as As-sensitive, and PMT1 and J49 as As-tolerant strains.

At 12 h, viable counts of S119 were significantly reduced at 40 and 50 µM As(V), with a 67.6% decrease at 50 µM relative to the control (Figure 1A). J260 did not change consistently with increasing As(V) concentration: viable counts were reduced at 10 µM, did not differ from the control at 20–40 µM, and showed the largest decrease at 50 µM, reaching a 79.1% reduction relative to the control (Figure 1A). In PMT1 and J49, viable counts were significantly reduced from 1 mM onwards. At 4 mM, they decreased by 84.5% and 94.0%, respectively, relative to their controls (Figure 1B). Despite these reductions, both strains maintained viable populations at an As(V) concentration 80-fold higher than the maximum tested for S119 and J260.

### 2.2. Plant Growth-Promoting Traits Under As(V) Exposure

Diazotrophic growth capacity, evidenced by the formation of a subsurface pellicle in semisolid nitrogen-free bromothymol blue (NFb) medium, was maintained in all four strains under As(V) exposure (Table 1). Siderophore production was not detected in S119 under either condition. In contrast, J49, PMT1, and J260 produced siderophores under the control, but this capacity was no longer detected under As(V) exposure (Table 1).

Phosphate-solubilization halo diameter was significantly affected by previous As(V) exposure in all four strains, although the direction of the response was strain-dependent. Compared with their respective controls, halo diameter decreased by 30.0% in J49, 47.4% in PMT1 and 34.6% in J260, whereas it increased by 45.8% in S119 (Table 1).

### 2.3. Quantification of Phosphate Solubilization and Cell-Associated As Concentration Under Strain-Specific As(V) Exposure

Under control conditions, soluble P concentrations differed significantly among strains, with the highest value in S119 and the lowest in J49, whereas PMT1 and J260 showed intermediate values that did not differ significantly from either strain. Under As(V) exposure, J49 and PMT1 showed the highest soluble P concentrations, followed by S119, whereas J260 showed the lowest value. Exposure to the respective strain-specific As(V) concentrations significantly increased soluble P concentration from 30.2 to 72.0 µg P/mL in J49 and from 33.2 to 80.6 µg P/mL in PMT1. No significant change was detected in S119, whereas J260 showed a significant decrease from 39.8 to 16.6 µg P/mL (Figure 2A).

Under the respective strain-specific exposure conditions, cell-associated As ranged from 115–142 µg As/g DW in J49 and PMT1 and from 66–74 µg As/g DW in S119 and J260 (Figure 2B). Because the exposed strains received different As(V) concentrations, these values describe the cell-associated As burden under the selected exposure conditions and do not constitute an equal-dose comparison of intrinsic accumulation capacity (Figure 2B).

### 2.4. Bacterial Oxidative and Antioxidant Responses

Based on their contrasting viability under As(V) exposure, the As-tolerant Enterobacter sp. J49 and the As-sensitive *Serratia* sp. S119 were selected for biochemical analysis at their respective strain-specific As(V) concentrations. Arsenate reductase activity was detected in both strains under control conditions and increased significantly following As(V) exposure, by 5.4-fold in J49 and 6.7-fold in S119 (Figure 3A).

Relative to their corresponding controls, H_2_O_2_ content increased significantly under As(V) exposure in both strains. Lipid peroxidation, assessed as thiobarbituric acid-reactive substances (TBARS) content, increased significantly in S119 by 3.3-fold, whereas no significant change was detected in J49 (Figure 3B,C).

SOD activity increased approximately fourfold in both strains following As(V) exposure. GPX and GST activities did not differ between control and As(V)-exposed J49 cultures, whereas both activities increased significantly in S119 by 206.9% and 311.2%, respectively (Figure 3D–F).

### 2.5. Effects of P Availability, PS-PGPB Coinoculation and As(V) Exposure on Peanut Growth and Nodulation

A significant Treatment × As(V) interaction was detected for shoot length (Figure 4A). In the absence of As(V), shoot length did not differ among treatments. Under As(V) exposure, plants receiving the P-free treatment showed significantly shorter shoots than those supplied with tricalcium phosphate (PTC), soluble P or PTC + S119, but did not differ from plants receiving PTC + J49. Comparisons between As(V) conditions within each treatment showed that shoot length decreased significantly only in the P-free treatment (29.7%) and PTC + S119 (20.5%), whereas no significant changes were detected in PTC, soluble P or PTC + J49.

Root length also showed a significant Treatment × As(V) interaction (Figure 4C), with no differences among treatments in the absence of As(V). Under As(V) exposure, the P-free treatment produced the shortest roots, whereas PTC showed the highest root length. Soluble P showed intermediate values that did not differ significantly from PTC or either treatment coinoculated with a PS-PGPB. PTC + S119 and PTC + J49 treatments did not differ significantly from each other. Treatment comparisons indicated that As(V) reduced root length in the P-free treatment (69.5%), PTC + S119 (34.4%) and PTC + J49 (51.3%), whereas no statistically significant reductions were detected in the PTC or soluble-P treatments.

The Treatment × As(V) interaction was not significant for shoot dry weight (Figure 4B). Across As(V) conditions, shoot dry weight was highest in plants coinoculated with J49 (PTC + J49), although it did not differ significantly from PTC + S119. The P-free treatment showed the lowest shoot dry weight and did not differ from soluble P. PTC showed intermediate values, overlapping with both PTC + S119 and soluble P. Independently of treatment, As(V) exposure reduced shoot dry weight by 13.3% (*p* = 0.0009). No statistically significant effect of As(V) exposure (*p* = 0.4557) or the Treatment × As(V) interaction was detected for root dry weight (Figure 4D). Across As(V) conditions, root dry weight was significantly lower in the P-free treatment than in the other four treatments, which did not differ significantly from one another.

The Treatment × As(V) interaction was not significant for either nodule number or nodule dry weight (Figure 4E,F). Independently of treatment, As(V) exposure reduced nodule number by 33% (*p* < 0.0001) and nodule dry weight by 30% (*p* = 0.0001). Across As(V) conditions, nodule number was lowest in plants supplied with soluble P, differing significantly from the P-free and PTC + J49 treatments, but not from PTC or PTC + S119. Nodule dry weight did not differ significantly among treatments.

For panels B, D, E, and F, the Treatment × As(V) interaction was not significant. Lowercase letters therefore indicate differences among marginal growth-condition means averaged across As(V) levels; the overall As(V) effect is reported within each panel. Mean comparisons were performed using Tukey’s test or heteroscedasticity-robust Holm-adjusted comparisons, as appropriate (*p* < 0.05).

### 2.6. Effects of As(V) Exposure and PS-PGPB Coinoculation on Shoot N Content and P Concentration

As(V) exposure significantly decreased shoot N content in all treatments except PTC, where it increased significantly (Table 2). Under control conditions, shoot N content was highest in PTC + J49, followed by soluble P and PTC + S119, which did not differ significantly from each other. The P-free treatment showed lower values, while PTC had the lowest N content. Under As(V) exposure, this pattern changed: PTC showed the highest N content, followed by PTC + J49 and then soluble P, whereas the P-free and PTC + S119 treatments showed the lowest values and did not differ significantly from each other. The corresponding individual shoot N concentrations are provided in Supplementary Data S1, allowing concentration-based responses to be distinguished from whole-shoot N accumulation. As(V) exposure significantly decreased shoot P concentration in PTC but increased it in the soluble-P treatment, whereas no significant changes were detected in the P-free, PTC + S119 or PTC + J49 treatments (Table 2).

Under As(V) exposure, plants supplied with soluble P or coinoculated with either strain showed the highest shoot P concentrations, with values significantly higher than those in the PTC and the P-free treatment.

### 2.7. Arsenic Accumulation and Translocation in Peanut Plants

Root As concentration did not differ significantly among the five treatments (Table 3). Shoot As concentration was also similar among the P-free, PTC and soluble P treatments, whereas As was not detected in shoots of either treatment coinoculated with a PS-PGPB.

Nodule As concentration differed significantly among treatments with detectable As, being highest in PTC (73.62 µg As/g DW), followed by PTC + J49 (57.43 µg As/g DW) and soluble P (15.59 µg As/g DW). As was not detected in nodules from the P-free or PTC + S119 treatments.

The translocation factor did not differ significantly among the P-free, PTC and soluble P treatments, with values of 0.28, 0.22 and 0.22, respectively. TF was not calculated for either treatment coinoculated with a PS-PGPB because shoot As was not detected.

## 3. Discussion

Beyond its direct impact on human health, As exposure can also affect crop plants and their associated rhizospheric microorganisms, potentially altering plant–microorganism interactions and impairing crop performance [7,20]. Moreover, the accumulation of As in edible plant tissues represents an additional route of exposure for animals and humans through the food chain, including the potential contamination of peanut kernels [21,22]. Against this regional background, groundwater As contamination in peanut-producing areas of Córdoba represents a realistic route of metalloid entry into irrigated agricultural systems [3,4]. Understanding the responses of rhizospheric microorganisms, plants, and their interactions to As is therefore essential for developing environmentally sustainable strategies to mitigate the effects of this environmental contaminant.

In this context, native PGPB constitute valuable biological resources for developing microbial inoculants adapted to local agricultural conditions, particularly in soils affected by nutrient limitations, such as low P availability, and by naturally occurring contaminants such as As. Indeed, field inoculation with PGPB has been shown to increase rice yield and improve nutrient availability in the rhizosphere, including available P [23]. The potential of this approach also extends to As-affected environments: inoculation with As-resistant PGPB increased rice grain yield under both greenhouse and field conditions, although their effects on grain As accumulation varied among bacterial strains and environmental conditions [24]. However, compared with the peanut–*Bradyrhizobium* sp. symbiosis, the capacity of non-rhizobial peanut-associated PGPB to function under As stress remains poorly explored. PS-PGPB strains J49, S119, and J260 were originally isolated from peanut nodules within the same broad agricultural region [25]. This shared origin, however, does not imply a uniform response, as bacterial tolerance can vary among strains depending on As speciation, exposure concentration, and the physiological mechanisms involved [15,16,17]. This variability was evident in the present study: J49 and PMT1 sustained growth at millimolar As(V) concentrations, whereas S119 and J260 showed pronounced inhibition within the micromolar range. However, broad classification as tolerant or sensitive did not fully capture the strain-specific viability and functional responses to As(V). J49 and S119 were therefore selected as contrasting native representatives for subsequent biochemical characterization and plant experiments because they differed markedly in their As(V)-tolerance thresholds while both retained phosphate-solubilizing activity under their respective strain-specific exposure conditions. Arsenate tolerance varies markedly within the genus *Enterobacter*: *E. cloacae* RSC3, isolated from an As-contaminated environment, tolerated substantially higher As(V) concentrations than J49 [26]. Thus, the tolerance of J49 at 4 mM As(V) represents a strain-specific phenotype and shows that substantial arsenate tolerance can also occur in bacteria without a documented history of exposure to heavily contaminated environments.

Beyond viability, strain-specific As(V) exposure differentially affected the operational detection of PGP traits. Diazotrophic growth remained detectable in all four strains under the selected exposure conditions. The CAS-agar assay was qualitative; therefore, the absence of an orange halo in J49, PMT1, and J260 is reported as siderophore production not detected under the assay conditions, rather than as evidence that the trait was abolished. Moreover, because the assay used strain-specific upper-range As(V) concentrations, these observations cannot be extrapolated to siderophore production in the rhizosphere at the 3 µM As(V) concentration used in the plant experiment. Phosphate-solubilization halo diameter was likewise treated as an operational, semiquantitative response because it integrates bacterial growth and radial diffusion after As(V) exposure. The quantitative soluble-P assay was therefore used as the primary measure of phosphate solubilization. Under the respective strain-specific exposure conditions, soluble P increased in J49 and PMT1, showed no statistically significant change in S119, and decreased in J260. These findings describe the responses observed under the in vitro exposure conditions and do not establish phosphate-solubilizing activity in the rhizosphere at 3 µM As(V).

Because culture pH, organic-acid production, and phosphatase activity were not measured, the mechanism underlying the observed changes in soluble P cannot be resolved. Metabolic remodeling associated with altered phosphate homeostasis, such as that reported by Zhuang et al. [27], provides a possible physiological context, but this mechanism was not tested in the present study. The detection of cell-associated As is consistent with previous reports of arsenic binding, uptake, and retention in resistant rhizobacteria [16,26,28]. Because the strains were exposed to different As(V) concentrations selected according to their respective tolerance ranges, the absolute cell-associated As values do not allow direct comparison of intrinsic accumulation capacity across strains. Rather, they represent the cell-associated As burden sustained by each strain while remaining viable and maintaining P-solubilizing activity. Total As in the bacterial pellets may comprise both intracellular and cell-envelope-bound fractions.

Arsenate reductase constitutes the entry point of the canonical intracellular As(V)-detoxification pathway, reducing arsenate to arsenite before its extrusion from the cell [29]. Unlike general antioxidant enzymes such as SOD, arsenate reductases are specialized components of arsenic-resistance systems whose occurrence and organization vary considerably among prokaryotic genomes [30]. Their functional detection in J49 and S119 therefore cannot be inferred solely from taxonomic identity and, to our knowledge, has not previously been reported in either strain. Moreover, the marked increase in arsenate reductase activity under As(V) exposure indicates that both strains enhanced their arsenate-reducing capacity. This reductive step is not metabolically neutral because it produces As(III), a more thiol-reactive species whose effective detoxification requires subsequent efflux and coordination with cellular redox metabolism. The comparable increase in arsenate reductase activity in J49 and S119 indicates that both strains share this response to As(V). However, their markedly different tolerance thresholds show that arsenate reduction alone does not determine the overall tolerance phenotype, consistent with Armendariz et al. [16], who observed similar As(V)-reducing capacities in two PGPB strains with contrasting metalloid sensitivities. Under their respective strain-specific exposure conditions, J49 and S119 showed different within-strain redox responses to As(V). Relative to their corresponding controls, H_2_O_2_ content and SOD activity increased significantly in both strains, whereas lipid peroxidation increased only in S119. This strain also showed significant increases in GPX and GST activities, whereas these activities remained unchanged in J49. Because arsenate reduction is closely connected to thiol and glutathione metabolism, the induction of GPX and GST in S119 points to a glutathione-dependent component of its response to As(V). Indeed, a bacterial GST has been shown to reduce As(V) directly and confer arsenate resistance in *Escherichia coli* [31], providing a mechanistic framework for interpreting the strong GST response observed in S119. Under the selected 50 µM As(V) exposure, S119 showed induction of multiple antioxidant and glutathione-related responses together with increased lipid peroxidation. Under the selected 4 mM As(V) exposure, J49 showed increased arsenate reductase and SOD activities without significant changes in GPX, GST, or lipid peroxidation. A comparable physiological contrast was previously reported in peanut-associated *Bradyrhizobium* strains: the As-sensitive SEMIA6144 developed oxidative damage despite activating antioxidant defenses, whereas the tolerant C-145 limited oxidative damage without broadly inducing its antioxidant machinery [32]. This previous observation provides a useful physiological context. In the present study, under their respective strain-specific exposure conditions, J49 showed no detectable increase in lipid peroxidation, whereas S119 showed increased oxidative damage. However, because J49 and S119 were exposed to different As(V) concentrations selected according to their respective growth-response ranges, strain identity, absolute As(V) concentration, and stress intensity relative to each strain’s tolerance could not be separated. The present findings therefore describe within-strain redox responses to the selected exposure conditions but do not demonstrate distinct mechanistic strategies of As(V) tolerance. A direct mechanistic comparison would require a common-dose design or dose–response analysis. The distinct enzymatic profiles reported in other As-tolerant bacteria further indicate that bacterial As tolerance has no universal antioxidant signature [33,34]. In J49 and S119, the relevant distinction was therefore the observed outcome of the response, the absence or occurrence of oxidative damage, rather than the number or direction of the antioxidant enzymes modulated. Nevertheless, the induction of arsenate reductase was demonstrated under strain-specific pure-culture exposure conditions and does not establish that either strain increased As(III) production or release in the rhizosphere at 3 µM As(V). Because As speciation was not determined in the bacterial medium, rhizosphere, roots, nodules, or shoots, the present results do not establish whether coinoculation altered the relative abundance or mobility of As(V) and As(III) in the plant–substrate system. The observed plant response should therefore be interpreted specifically as restriction of detectable total As translocation to shoots, rather than as evidence of As detoxification or reduced chemical risk throughout the plant system. Direct HPLC–ICP-MS analysis of As species in the rhizosphere solution and plant organs would be required to resolve this question.

In planta, P availability modulated the growth response of peanut plants to As(V), particularly at the level of organ elongation. The strongest inhibition of root elongation occurred in the P-free treatment, whereas soluble P and PTC maintained the highest root lengths under As exposure. J49 and S119 produced distinct patterns of organ elongation under As(V), whereas neither strain provided treatment-specific protection of shoot biomass. Among the coinoculated treatments, S119 showed the clearest attenuation of root-growth inhibition, although its response did not differ significantly from that observed with J49, while shoot length was maintained only in J49-coinoculated plants. These contrasting responses indicate that the effects of PS-PGPB coinoculation on plant growth were strain- and organ-dependent.

The beneficial effects of non-rhizobial PS-PGPB on peanut performance have been demonstrated across different agricultural systems. Phosphate-solubilizing and native P-mobilizing bacteria improved nutrient acquisition, nodulation, biomass, and yield under controlled and field conditions, while root- and nodule-associated endophytes enhanced plant growth and symbiotic performance either alone or in combination with rhizobia [11,12,35,36].

Evidence for this functional complementation under As exposure remains considerably more limited. Martos et al. [19] showed that an amendment combining spent mushroom substrate with *Pseudomonas fluorescens* strain 1.2 supported peanut biomass under 20 µM As(V), while Sathya et al. [37] reported improved peanut growth and chlorophyll content following inoculation with As-tolerant PGPB in soil amended with 200 mg As/kg. Together, these studies demonstrate the potential of beneficial bacteria to sustain peanut performance under As stress. The present study advances this framework by examining the contribution of native PS-PGPB in association with a defined microsymbiont, *Bradyrhizobium* sp. SEMIA6144, and by integrating their effects on plant growth, nodulation, nutrient acquisition, and organ-specific As distribution.

Nodulation, however, showed a more uniform response to the metalloid, as As(V) reduced both nodule number and nodule dry weight independently of P availability or PS-PGPB coinoculation. Thus, neither P supplementation nor the additional bacterial partner prevented the inhibitory effect of As(V) on the peanut–*Bradyrhizobium* symbiosis. A similar response was previously observed at the same As(V) concentration, where nodule number and biomass decreased in peanut plants inoculated with either SEMIA6144 or C-145 [18]. The persistence of this effect despite the maintenance of root biomass indicates that improved P nutrition supported vegetative growth but was insufficient to preserve nodulation completely under As(V) exposure.

As(V) altered shoot N accumulation according to P availability and PS-PGPB coinoculation. Because the nutrient solution contained no combined N and all plants were inoculated with *Bradyrhizobium* sp. SEMIA6144, shoot N accumulation largely reflected the functioning of the peanut–rhizobium symbiosis. Its general decline under As(V) was accompanied by reductions in nodule number and biomass. Nevertheless, shoot N accumulation was not proportional to nodule number or biomass. Under control conditions, coinoculation with either PS-PGPB increased shoot N content relative to PTC alone, with the highest value observed in J49-coinoculated plants. This benefit was not maintained under As(V): shoot N remained higher with J49 than with S119, but both coinoculated treatments showed lower values than PTC alone. Thus, J49 and S119 differentially influenced plant N nutrition despite interacting with the same microsymbiont. Most importantly, both PS-PGPB maintained shoot P concentration under As(V) when PTC was the only external P source. Plants coinoculated with J49 or S119 reached shoot P concentrations comparable to those of plants supplied with soluble P and significantly higher than those of plants receiving PTC without an additional PS-PGPB. In the latter treatment, shoot P decreased under As(V), indicating that plants did not efficiently acquire P from the insoluble source in the absence of a PS-PGPB partner. This contrast is consistent with competition between phosphate and its chemical analogue arsenate for cellular uptake and with a contribution of the coinoculation treatments to plant P acquisition in the PTC-supplied system. However, because rhizosphere P availability and pH were not measured, in situ PTC mobilization cannot be identified as the demonstrated mechanism. The absence of an As-induced decrease in the P-free treatment does not contradict this interpretation, because P concentration was already constrained by the lack of an external P source. Because the P-free treatment received no external phosphate, the P detected in shoots likely derived primarily from remobilization of endogenous P reserves from the seed and cotyledons during seedling establishment. Similar improvements in peanut P acquisition following bacterial mobilization of poorly available inorganic P pools have been reported previously [11,12].

The relationship between the pure-culture and plant experiments further highlights the context dependence of bacterial PGP activity. Although As(V) increased soluble P concentrations in J49 cultures but produced no significant change in S119, both strains maintained similarly high shoot P concentrations when associated with peanut and *Bradyrhizobium* sp. SEMIA6144. The pure-culture assays established the contrasting physiological responses of J49 and S119 to As(V), whereas the plant experiment confirmed that both strains retained their capacity to support P nutrition within the complete peanut–PS-PGPB–*Bradyrhizobium* association. These complementary levels of analysis link the bacterial response to As(V) with its functional expression in planta. Nevertheless, comparable maintenance of P nutrition did not result in an equivalent response in shoot N, which remained higher with J49 than with S119. This uncoupling indicates that the PS-PGPB coinoculation treatments supported P nutrition, whereas the broader performance of the peanut–SEMIA6144 symbiosis also reflected strain-specific effects on N nutrition under As(V). Both J49 and S119 were originally isolated as peanut nodule endophytes. Moreover, previous field experiments using the same 1:1 J49–SEMIA6144 inoculation ratio confirmed the persistence of J49 in rhizosphere soil through harvest by selective plating and ERIC-PCR [14]. Nevertheless, persistence of either strain, rhizosphere P availability, and pH were not quantified under the specific As(V) exposure conditions of the present study.

The clearest effect of PS-PGPB coinoculation on As partitioning was the restriction of detectable total As transfer to the aerial tissues. Root As concentrations did not differ significantly among P treatments, whereas As was not detected in the shoots of plants coinoculated with either J49 or S119. This organ-specific pattern indicates that both PS-PGPB modified As partitioning within the plant, limiting root-to-shoot translocation rather than uniformly reducing root As accumulation. Moreover, this restriction occurred while shoot P concentration was maintained at levels comparable to those achieved with soluble P, showing that J49 and S119 preserved P acquisition while selectively limiting metalloid transfer to the shoot.

The nodule data further revealed that the two PS-PGPB produced different belowground As distribution patterns. In J49-coinoculated plants, As was not detected in shoots but accumulated in nodules, where its concentration reached 57.43 µg As g^−1^ DW. This pattern is consistent with metalloid retention in belowground symbiotic tissues. In contrast, As was not detected in either shoots or nodules of S119-coinoculated plants, despite the absence of significant differences in root As concentration among treatments. Thus, S119 coinoculation was associated with the absence of detectable As in both nodules and shoots, whereas J49 coinoculation produced a pattern of As retention within the root–nodule compartment. Despite these distinct belowground distribution patterns, both associations converged on the same agronomically relevant outcome: restricted detectable total As translocation to shoots and no detectable As in the shoot.

The behaviour of J49 is particularly informative when compared with plants supplied with PTC alone. Both treatments contained the same insoluble P source, but nodule As accumulation in the absence of an additional PS-PGPB was accompanied by detectable As in the shoot. In contrast, relative to plants supplied with PTC alone, J49 coinoculation maintained shoot P concentration, reduced nodule As accumulation, and prevented detectable As transfer to the aerial tissues, despite no statistically significant difference in root As concentration. This contrast is consistent with a contribution of J49 coinoculation to plant P acquisition in the PTC-supplied system, potentially contributing to the restriction of metalloid translocation beyond the belowground compartments. These findings extend previous observations showing that the bacterial partner influences As partitioning in peanut. At the same As(V) concentration, inoculation with *Bradyrhizobium* sp. SEMIA6144 resulted in greater As retention in roots and nodules and a lower translocation factor than inoculation with strain C-145, which better sustained nodulation and N nutrition but allowed greater As movement towards the shoot [18]. SEMIA6144 was therefore selected for the present study because of its previously demonstrated capacity to restrict As translocation, whereas coinoculation with J49 or S119 was intended to reinforce plant growth and nutrient acquisition. The absence of detectable shoot As in both coinoculated treatments indicates that the inoculum combinations containing SEMIA6144 and either J49 or S119 preserved the phytostabilizing outcome previously associated with SEMIA6144. Indeed, coinoculation may have reinforced this response, since shoot As remained detectable in plants inoculated with SEMIA6144 alone. Because organ As contents and a whole-plant As mass balance were not determined, the present conclusions refer to organ As concentrations and detectable translocation rather than to total plant As uptake. Alongside this convergent restriction of shoot As, shoot N accumulation and nodule As revealed additional treatment-specific functional differences. Under As(V), PTC alone produced the highest shoot N content, whereas both coinoculated treatments maintained higher shoot P concentrations but lower shoot N than PTC alone, with J49 retaining more shoot N than S119. Shoot N did not parallel nodule number or dry weight, indicating that morphological measures of nodulation alone did not reflect symbiotic N performance; moreover, shoot N expressed as mg per plant was jointly determined by shoot biomass and tissue N concentration. Nodule As also showed treatment-specific partitioning that was not explained by root As concentration or nodule size: P-free plants contained As in roots and shoots but remained below the detection limit in nodules, whereas PTC-treated plants accumulated high nodule As despite the absence of a significant difference in root As concentration and without a significantly larger nodule compartment. These patterns may reflect differences in PTC solubilization and Ca–P–As chemistry, nodule physiological status, source–sink relations, belowground As retention, and the export or allocation of fixed N compounds. However, because rhizosphere pH, soluble Ca and As, nodule P content, nitrogenase activity, xylem metabolites, and As speciation were not measured, the relative contribution of these processes cannot be resolved.

The contrast between J49 and S119 demonstrates why maximal bacterial As(V) tolerance in pure culture should not be used as the sole criterion for inoculant selection. In a previous study, *Pseudomonas fluorescens* 1.2 was selected over J49 because it tolerated a higher maximum As(V) concentration in vitro; however, the 20 µM subsequently applied to plants was within the viability range of both strains, preventing evaluation of whether their different tolerance thresholds predicted their performance in planta [19]. The present study instead compared two PS-PGPB with contrasting tolerance phenotypes at a common, environmentally relevant As(V) concentration. Although J49 and S119 displayed markedly different tolerance thresholds, both coinoculation treatments maintained shoot P nutrition and restricted shoot As to below the method detection limit at 3 µM As(V). Their contrasting effects on growth, N nutrition, and belowground As partitioning nevertheless demonstrate that plant-level performance emerges from the complete strain–plant–rhizobium interaction. The present experiment was conducted with a single peanut cultivar and rhizobial strain in sterilized sand–perlite, at one As(V) concentration, and was terminated during vegetative development. It therefore does not reproduce interactions with competing soil microbiota or the sorption–desorption processes that govern P and As availability under field conditions. To our knowledge, this is the first report showing that coinoculation of peanut with native PS-PGPB and *Bradyrhizobium* sp. SEMIA6144 can restrict detectable total As translocation to shoots under controlled As(V) exposure. Further studies under field conditions and throughout reproductive development are required to determine whether this response extends to pods and kernels.

## 4. Materials and Methods

### 4.1. Bacterial Strains

*Enterobacter* sp. J49, *Serratia* sp. S119 and *Serratia* sp. J260 were isolated from root nodules of peanut (*Arachis hypogaea* L.) plants grown in the peanut-producing area of Córdoba Province, Argentina, from a collection of 433 bacterial isolates [25,38]. *Pseudomonas pergaminensis* PMT1 is a PS strain used in the commercial inoculant formulation RIZOFOS^®^ in Argentina [10]. Strains were routinely maintained on Luria–Bertani (LB) agar plates at 4 °C. For long-term storage, aliquots from late-exponential-phase cultures were supplemented with sterile glycerol to a final concentration of 40% (*v*/*v*) and stored at −20 °C.

### 4.2. Effect of Arsenate on Bacterial Growth and Viability

Bacterial cultures were grown in 25 mL of TY medium [39] at 26 °C on an orbital shaker at 150 rpm. Cultures previously grown in As-free TY medium were used to inoculate the experimental cultures to an initial optical density of 0.1. Arsenic was supplied as arsenate [As(V)] using sodium arsenate heptahydrate (Na_2_HAsO_4_·7H_2_O), and cultures without As(V) were used as controls.

Bacterial growth was initially screened in all four strains using progressively increasing As(V) concentrations up to 4 mM. The concentration range was subsequently adjusted according to the growth response of each strain, allowing time-course growth to be characterized under conditions that produced measurable inhibition without complete loss of viability. Accordingly, J49 and PMT1 were evaluated at 0, 1, 2, 3, and 4 mM As(V), whereas S119 and J260 were evaluated at 0, 10, 20, 30, 40, and 50 µM As(V). These strain-specific concentration ranges were designed to characterize upper tolerance limits in pure culture, whereas the plant experiment used 3 µM As(V) to represent an environmentally relevant exposure.

Samples were collected at 2-h intervals over a 12-h incubation period. Cell viability was determined using the microdroplet method described by Somasegaran and Hoben [40] and expressed as colony-forming units per millilitre (CFU/mL). Three independent cultures were analysed for each strain and As(V) concentration.

### 4.3. Plant Growth-Promoting Traits Under Arsenate Exposure

Before each assay, bacterial strains were grown in As(V)-free medium, and the resulting cultures were adjusted to an optical density at 620 nm (OD_620_) of 0.1. Standardized inocula were subsequently transferred to the assay-specific media prepared either without As(V) or supplemented with the strain-specific As(V) concentration selected from the preceding growth analysis. The selected concentrations were 4 mM As(V) for *Enterobacter* sp. J49 and *P. pergaminensis* PMT1, and 50 µM As(V) for *Serratia* sp. S119 and *Serratia* sp. J260. These concentrations represented the highest tested As(V) levels compatible with sufficient bacterial growth to conduct the subsequent physiological assays.

#### 4.3.1. Potential Diazotrophic Growth

Potential diazotrophic growth was evaluated using nitrogen-free semisolid malate medium (NFb) according to Döbereiner et al. [41]. Standardized bacterial inocula were transferred to solid NFb medium prepared either without As(V) or supplemented with the corresponding strain-specific As(V) concentration. Plates were incubated at 28 °C until bacterial growth was established. Growth from each experimental condition was subsequently transferred to tubes containing 4 mL of the corresponding semisolid NFb medium, prepared with 3 g/L agar and adjusted to pH 6.5. Tubes were incubated at 28 °C for 7 days. The formation of a subsurface pellicle was recorded as a positive response for potential diazotrophic growth. *Azospirillum argentinense* Az39^T^ was included as a positive control.

#### 4.3.2. Siderophore Production

Siderophore production was qualitatively evaluated on Chrome Azurol S (CAS) agar according to Schwyn and Neilands [42]. Standardized bacterial inocula were cultured on CAS medium prepared either without As(V) or supplemented with the corresponding strain-specific As(V) concentration. Plates were incubated at 28 °C for 7 days. Siderophore production was identified by the formation of an orange halo around the bacterial growth, resulting from iron removal from the blue Fe–CAS complex.

#### 4.3.3. Phosphate Solubilization

Phosphate-solubilizing capacity was evaluated qualitatively and quantitatively using National Botanical Research Institute’s phosphate growth medium (NBRIP), following Mehta and Nautiyal [43], with modifications to incorporate strain-specific As(V) exposure.

Bacterial cultures were grown for 24 h and centrifuged, and the resulting pellets were resuspended in 1 mL of nutrient broth. An appropriate volume of each suspension was then used to inoculate 10 mL of liquid NBRIP medium, prepared either without As(V) or with the corresponding strain-specific As(V) concentration, to obtain an initial OD_620_ of 0.1–0.2. Cultures were incubated for 12 h at 26 °C on an orbital shaker at 150 rpm.

For the qualitative assay, 10-µL aliquots of the 12-h cultures were inoculated onto As(V)-free solid NBRIP medium. Plates were incubated at 26 °C for 5–7 days. Phosphate solubilization was identified by the formation of a transparent halo around bacterial growth, and halo diameter was measured and expressed in centimetres. Eight replicates were analysed for each strain and pre-exposure condition.

For the quantitative assay, aliquots of the same 12-h liquid cultures were centrifuged at 10,000× *g* for 12 min to remove bacterial cells and insoluble phosphate. Soluble orthophosphate in the resulting supernatants was determined spectrophotometrically at 660 nm using the modified colorimetric method of Fiske and Subbarow [44]. Uninoculated NBRIP medium subjected to the same experimental conditions was used as the reaction blank. Phosphate-solubilizing capacity was expressed as µg soluble P/mL. Between four and seven independent replicates were analysed per strain and experimental condition.

### 4.4. Cell-Associated Arsenic Concentration

Bacterial cultures were grown for 12 h in TY medium supplemented with the strain-specific As(V) concentration selected in Section 4.3. Cultures were centrifuged at 12,000× *g* for 10 min, and the resulting bacterial pellets were washed to remove residual As-containing culture medium. Pellets were oven-dried at 65 °C for approximately 48 h, until constant weight, and then weighed.

Dried bacterial biomass was digested using an acid-oxidizing mixture of H_2_O, HNO_3_, and 30% H_2_O_2_ (5:3:2), adapted from Sobrino-Plata et al. [45]. Digestion was performed under high-pressure conditions using a microwave digestion system equipped with an HPR1000/10S segmented rotor. The resulting digests were brought to a final volume of 4 mL with bidistilled water. Arsenic concentration was determined using an ELAN DRC-e inductively coupled plasma mass spectrometer (ICP-MS; PerkinElmer SCIEX, Thornhill, ON, Canada) and expressed as µg As/g DW. Three independent biological replicates were analysed for each strain.

### 4.5. Selection of Representative Strains and Arsenate Reductase Activity

Following the initial screening, *Enterobacter* sp. J49 and *Serratia* sp. S119 were selected for subsequent biochemical characterization and plant experiments because they represented markedly contrasting As(V)-tolerance thresholds while both retained phosphate-solubilizing activity under their respective strain-specific As(V) exposure conditions. J260 was not advanced because its phosphate-solubilizing activity decreased significantly under As(V), whereas PMT1 was included in the screening as a commercial reference strain and fell outside the subsequent focus on native peanut-associated bacteria. For these assays, J49 and S119 were exposed to 4 mM and 50 µM As(V), respectively, according to the strain-specific concentrations established in Section 4.3. Cultures grown without As(V) were used as controls.

After 12 h of As(V) exposure, bacterial cultures were centrifuged at 10,000× *g* for 10 min at 4 °C. The resulting pellets were washed twice with sterile physiological saline. For arsenate reductase, superoxide dismutase (SOD), glutathione peroxidase (GPX), and glutathione S-transferase (GST) determinations, pellets were resuspended in 1 mL of 50 mM potassium phosphate buffer. For hydrogen peroxide and lipid peroxidation determinations, pellets were resuspended in 1 mL of 10% (*w*/*v*) trichloroacetic acid (TCA), and pellet fresh weight was recorded before extraction. Cell suspensions or homogenates were sonicated for 2 min at an amplitude setting of 80 using 6-s pulses and subsequently centrifuged under the same conditions. The resulting supernatants were used as cell-free extracts for the corresponding assays.

Arsenate reductase activity was determined according to Anderson and Cook [46] by monitoring NADPH oxidation spectrophotometrically at 340 nm. One unit of arsenate reductase activity was defined as the amount of enzyme catalysing the oxidation of 1 µmol of NADPH per minute. Specific activity was expressed as U/mg protein. Endogenous NADPH oxidation was determined in parallel no-arsenic controls and subtracted from the arsenate-induced NADPH oxidation rate, following Anderson and Cook [46].

Normalization was selected according to the analytical endpoint. Enzyme activities were expressed per mg protein as specific activities. H_2_O_2_ content was normalized to the protein concentration determined in the parallel phosphate-buffer enzyme extract from the same biological replicate. TBARS, as an index of lipid oxidation associated with cellular biomass, was normalized to the fresh weight of the bacterial pellet recorded before extraction. The same normalization basis was applied to all strains and treatments within each assay, and absolute values were not compared across assays.

Protein concentration in the phosphate-buffer cell-free extracts was determined according to Bradford [47], using bovine serum albumin as the calibration standard.

### 4.6. Bacterial Oxidative Stress Markers

Cell-free extracts for hydrogen peroxide and lipid peroxidation determinations were prepared as described in Section 4.5, using 10% (*w*/*v*) trichloroacetic acid as the extraction medium.

Hydrogen peroxide content was determined according to Alexieva et al. [48]. Briefly, 160 µL of supernatant was mixed with 160 µL of 100 mM potassium phosphate buffer (pH 6.8) and 680 µL of 1 M potassium iodide. The reaction mixture was incubated in darkness for 1 h, and absorbance was measured at 390 nm. Hydrogen peroxide content was calculated using a calibration curve prepared from a 1 mM H_2_O_2_ stock solution and expressed as nmol H_2_O_2_/mg protein.

Lipid peroxidation was estimated by determining thiobarbituric acid-reactive substances (TBARS), according to Heath and Packer [49]. Absorbance was measured at 535 nm, and nonspecific absorbance was corrected by subtracting the absorbance recorded at 600 nm. Lipid peroxidation was expressed as nmol TBARS/g FW.

### 4.7. Selected Bacterial Antioxidant Enzyme Activities

Cell-free extracts for the determination of SOD, GPX, and GST activities were prepared as described in Section 4.5, using 50 mM potassium phosphate buffer as the extraction medium.

Superoxide dismutase activity was determined by measuring the inhibition of the photochemical reduction of nitroblue tetrazolium (NBT), according to Beauchamp and Fridovich [50], with modifications. The 1-mL reaction mixture contained 50 mM potassium phosphate buffer (pH 7.8), 0.54 µM EDTA, 75 µM NBT, 777 µM methionine, 4 µM riboflavin, and 5 µg of protein extract. Reaction mixtures were exposed to fluorescent light for 15 min, and absorbance was measured at 560 nm. One unit of SOD activity was defined as the amount of enzyme required to inhibit NBT reduction by 50%. Activity was expressed as U/mg protein.

Glutathione peroxidase activity was determined using the coupled assay described by Flohé and Günzler [51], with modifications. The assay contained 100 µL of enzyme extract, 500 µL of 0.1 M potassium phosphate buffer (pH 7.4), 100 µL of glutathione reductase corresponding to 0.24 U, and 20 µL of 10 mM reduced glutathione. The reaction was initiated by adding 100 µL of 1.5 mM NADPH and 100 µL of 10 mM H_2_O_2_. NADPH oxidation was monitored as the decrease in absorbance at 340 nm for 5 min. GPX activity was calculated from the decrease in absorbance at 340 nm resulting from NADPH oxidation and expressed as µmol NADPH oxidized/min/mg protein, using a molar extinction coefficient for NADPH of 6.2 L/mmol/cm. Glutathione S-transferase activity was determined according to Habig et al. [52], with modifications, by monitoring the conjugation of reduced glutathione with 1-chloro-2,4-dinitrobenzene (CDNB). The 1-mL reaction mixture contained 125 µL of 0.25 mM CDNB, 400 µL of 4 mM reduced glutathione, 200 µg of protein extract, and 0.1 M potassium phosphate buffer (pH 7.0). Enzyme activity was monitored continuously at 340 nm for 5 min at 25 °C. Activity was calculated using a molar extinction coefficient of 9.6 L/mmol/cm for the CDNB–GSH conjugate. GST activity was calculated from the rate of CDNB–GSH conjugate formation and expressed as µmol CDNB–GSH conjugate produced per min per mg protein (µmol CDNB–GSH conjugate/min/mg protein).

### 4.8. Plant Material, Growth Conditions, and Experimental Design in Microcosms Assay

Peanut (*Arachis hypogaea* L.) seeds of the commercial cultivar Granoleico were used. Seeds were surface-disinfected by immersion in 96% (*v*/*v*) ethanol for 30 s, followed by treatment with 6% (*v*/*v*) H_2_O_2_ for 15 min, according to Vincent [53]. After thorough rinsing with sterile distilled water, seeds were placed in sterile Petri dishes containing moistened cotton and filter paper and incubated at 28 °C in darkness for 72 h, until the radicles reached approximately 2–3 cm in length.

Pre-germinated seeds were transferred to modified Leonard jar assemblies consisting of two superimposed plastic containers. The upper container was filled with a sterile 2:1 (*v*/*v*) sand–perlite mixture and connected to the lower nutrient-solution reservoir by filter-paper wicks. The lower container contained a modified nitrogen-free Hoagland nutrient solution [54]. The liquid level was maintained throughout the experiment by periodically replenishing the corresponding nutrient solution. For plants assigned to the As(V) treatments, the solution was replenished while maintaining an As(V) concentration of 3 µM. Each Leonard jar contained a single plant and constituted an independent experimental unit. The number of biological replicates varied among determinations and is indicated in the corresponding tables and figure legends. Plants were assigned to five phosphorus and bacterial-inoculation treatments: (i) a P-free treatment, prepared by omitting the phosphorus source from the nutrient solution; (ii) insoluble phosphorus supplied as tricalcium phosphate (Ca_3_(PO_4_)_2_); (iii) soluble phosphorus supplied as K_2_HPO_4_ at a final concentration of 2 mM, prepared from a 1 M stock solution; (iv) PTC supplemented with *Serratia* sp. S119; and (v) PTC supplemented with *Enterobacter* sp. J49. Each treatment was evaluated in the absence or presence of 3 µM As(V), supplied as sodium arsenate heptahydrate (Na_2_HAsO_4_·7H_2_O), resulting in ten treatment combinations. The insoluble-P treatment received a single application of 3 mL per pot of a 16.1 mM Ca_3_(PO_4_)_2_ suspension (5 g/L), corresponding to approximately 15 mg PTC, or 3.0 mg total P, per pot.

At 7 days post-emergence, plants receiving only the symbiotic strain were inoculated at the root crown with 3 mL of a *Bradyrhizobium* sp. SEMIA6144 suspension containing approximately 1 × 10^8^ CFU/mL. For the coinoculation treatments, a total inoculum volume of 3 mL was applied, consisting of 1.5 mL of *Bradyrhizobium* sp. SEMIA6144 and 1.5 mL of either *Serratia* sp. S119 or *Enterobacter* sp. J49. Each bacterial suspension contained approximately 1 × 10^8^ CFU/mL. Plants were maintained in a controlled-environment growth chamber under a photon flux density of 200 µmol/m^2^/s, a 16 h light/8 h dark photoperiod, a temperature of 28 °C, and approximately 50% relative humidity. Plants were harvested after approximately 40 days, when they had reached the R1 stage, beginning flowering, according to the phenological scale described by Boote [55].

At harvest, shoots, roots, and nodules were separated. Shoot and root lengths were measured, with root length determined from the root collar to the tip of the longest root. Nodules were removed and counted. Plant fractions were subsequently dried at 70 °C until constant weight to determine shoot, root, and nodule dry weights.

The number of biological replicates differed among variables because of their specific sampling and material requirements. Morphological variables were recorded in larger sets of individual plants, whereas destructive biomass measurements and nutrient and organ-specific As determinations were performed on variable-specific analytical subsets. For some organ-specific analyses, the number of usable replicates was additionally constrained by the availability of sufficient dry material. For nodule As determination, dried nodules from multiple plants within each biological replicate were pooled to obtain the required mass for each analytical sample. Exact sample sizes for every treatment × As(V) × variable combination are provided in Table S2.

### 4.9. Plant Nitrogen Content and Phosphorus and Arsenic Concentrations in Plant Tissues

Shoot nitrogen content was determined using the modified Kjeldahl method described by Nelson and Sommers [56] and expressed as mg N/plant. Shoot phosphorus concentration was determined according to Jackson [57]. Dried plant material was digested with concentrated HNO_3_ for 48 h at room temperature, and P was quantified colorimetrically at 430 nm using the vanadate–molybdate reagent. Results were expressed as mg P/g DW.

Total As concentration was determined separately in shoots, roots, and nodules. Approximately 0.1 g of dried shoot or root material was used for digestion. For nodule analysis, dried nodules from multiple plants within each biological replicate were pooled to obtain 0.1 g DW per analytical sample. Samples were digested with ultrapure H_2_O, HNO_3_, and 30% H_2_O_2_ at a 5:3:2 ratio, following Sobrino-Plata et al. [45]. Digests were brought to a final volume of 10 mL with ultrapure water, and a 0.5-mL aliquot was subsequently diluted to 15 mL. Total As was determined using an ELAN DRC-e inductively coupled plasma mass spectrometer. The instrumental limits of detection and quantification for As were 0.5 and 2 µg/L, respectively. Based on the nominal sample mass of 0.1 g DW and the dilution procedure, the corresponding method LOD and LOQ were approximately 1.5 and 6 µg As/g DW, respectively. Arsenic concentrations were corrected for the 30-fold dilution and expressed as µg As/g DW. Samples in which As was not detected under the analytical conditions employed were reported as ND. The translocation factor (TF) was calculated as the ratio of shoot As concentration to root As concentration, according to Singh and Agrawal [58]. TF was not calculated when shoot As was not detected.

### 4.10. Statistical Analysis

Data are presented as means ± SE, with significance established at *p* < 0.05. Analyses were performed using InfoStat version 2020, except for the robust analyses described below. Homogeneity of variance was assessed from absolute residuals. Logarithmic, square-root or normal-score transformations were applied when required; analyses were conducted on transformed data, whereas untransformed means ± SE are reported.

Bacterial viability was analysed separately for each strain using generalized least-squares models fitted by REML, with As(V) concentration, time and their interaction as fixed effects. Temporal dependence among repeated measurements from the same culture was modelled using an AR(1) correlation structure. Significant interactions were followed by comparisons among concentrations within each sampling time using Tukey’s test. Because variance heterogeneity persisted for J260 at 12 h, this time point was analysed by Welch’s ANOVA followed by pairwise Welch comparisons with Holm-adjusted *p*-values. Viability data were expressed as log_10_ CFU/mL.

Continuous bacterial physiological and biochemical variables were analysed separately within each strain, and strains were compared within each exposure condition using Tukey’s test. Comparisons among exposed strains represent responses under their respective exposure conditions rather than equal-dose comparisons. Phosphate-solubilization halo diameter was analysed using a factorial model with HC3 heteroscedasticity-consistent standard errors and Holm-adjusted pairwise comparisons.

Plant variables were analysed using factorial models with P/inoculation treatment and As(V) exposure as fixed factors and Type III sums of squares. Significant interactions were examined through simple effects, whereas nonsignificant interactions were analysed using marginal means and the overall As(V) effect. Mean comparisons were performed using Tukey’s test. Root length and root dry weight, for which heteroscedasticity persisted after transformation, were analysed on untransformed data using HC3 robust standard errors and Holm-adjusted comparisons in Python 3.12 with statsmodels version 0.14.6. Arsenic concentrations in individual organs and the translocation factor, determined only in As(V)-exposed plants, were analysed separately by one-way ANOVA followed by Tukey’s test when the assumptions were met. For organ As analyses, measurements below the method detection limit were treated as left-censored observations, reported as ND, and not replaced by zero or another numerical surrogate.

## 5. Conclusions

This study demonstrates that, despite their contrasting upper-range As(V)-tolerance phenotypes and distinct redox responses under strain-specific in vitro exposures, both PS-PGPB strains produced a convergent response at 3 µM As(V): they sustained peanut P nutrition and restricted shoot As to below the method detection limit, while exerting strain-specific effects on plant growth, N nutrition, and belowground As partitioning. Effective inoculant selection for As-affected environments should therefore consider the integrated performance of the complete strain–plant–rhizobium association. These findings demonstrate that coinoculation of native PS-PGPB with *Bradyrhizobium* sp. SEMIA6144 offers a promising strategy for improving P acquisition from poorly available sources while restricting detectable total As translocation to shoots under the controlled conditions tested. Further studies are required to validate these effects under field conditions.

## Figures and Tables

**Figure 1:**
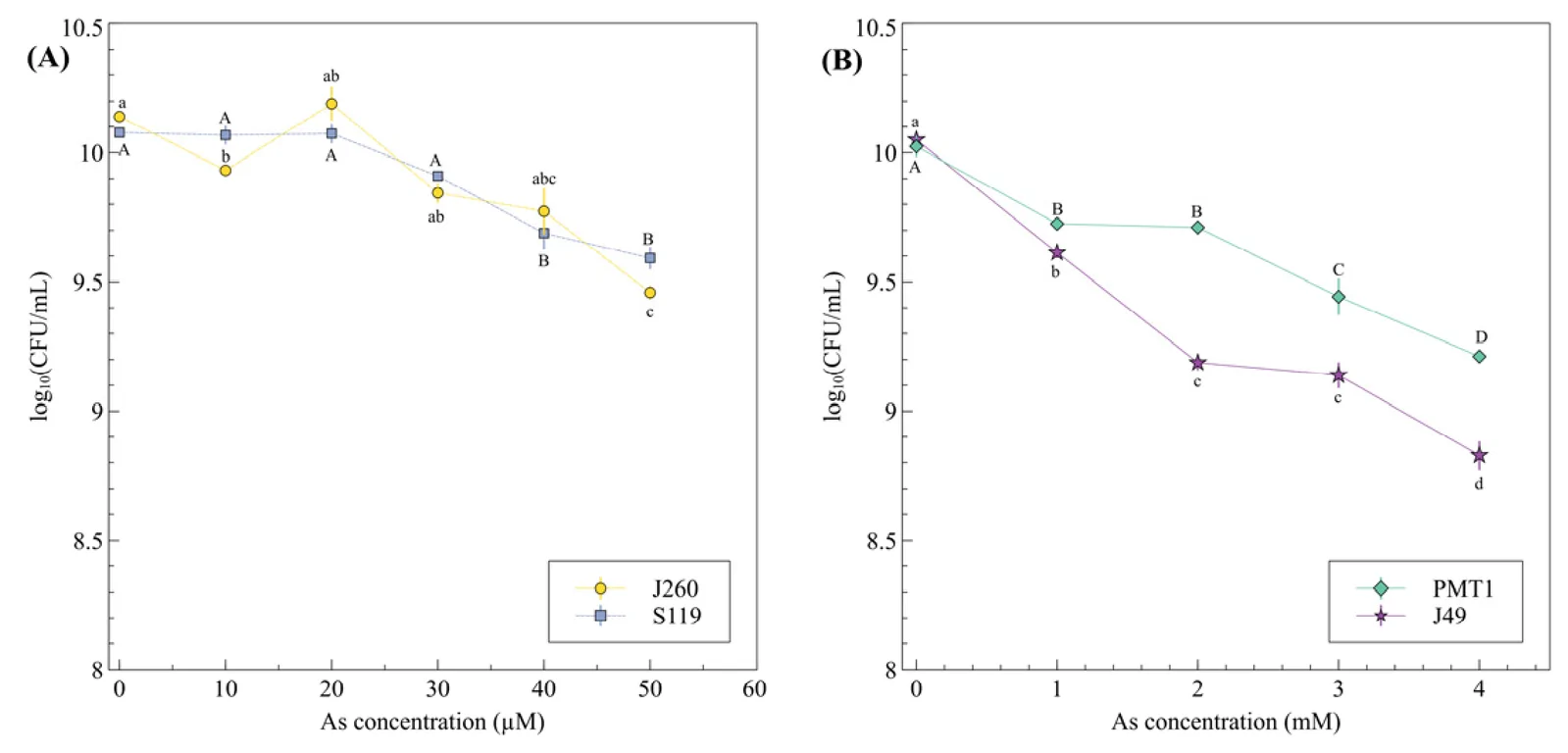
Effect of As(V) concentration on bacterial viable counts after 12 h of exposure. (**A**) J260 and S119 exposed to 0–50 µM As(V). (**B**) PMT1 and J49 exposed to 0–4 mM As(V). Values are presented as means ± SE (*n* = 3 biological replicates). Within each strain, different letters indicate significant differences among As(V) concentrations (*p* < 0.05), based on Tukey’s test, except for J260, for which comparisons were performed using Welch’s ANOVA followed by pairwise Welch comparisons with Holm-adjusted *p* values, due to persistent variance heterogeneity at 12 h. In panel (**A**), lowercase and uppercase letters correspond to J260 and S119, respectively; in panel (**B)**, uppercase and lowercase letters correspond to PMT1 and J49, respectively.

**Figure 2:**
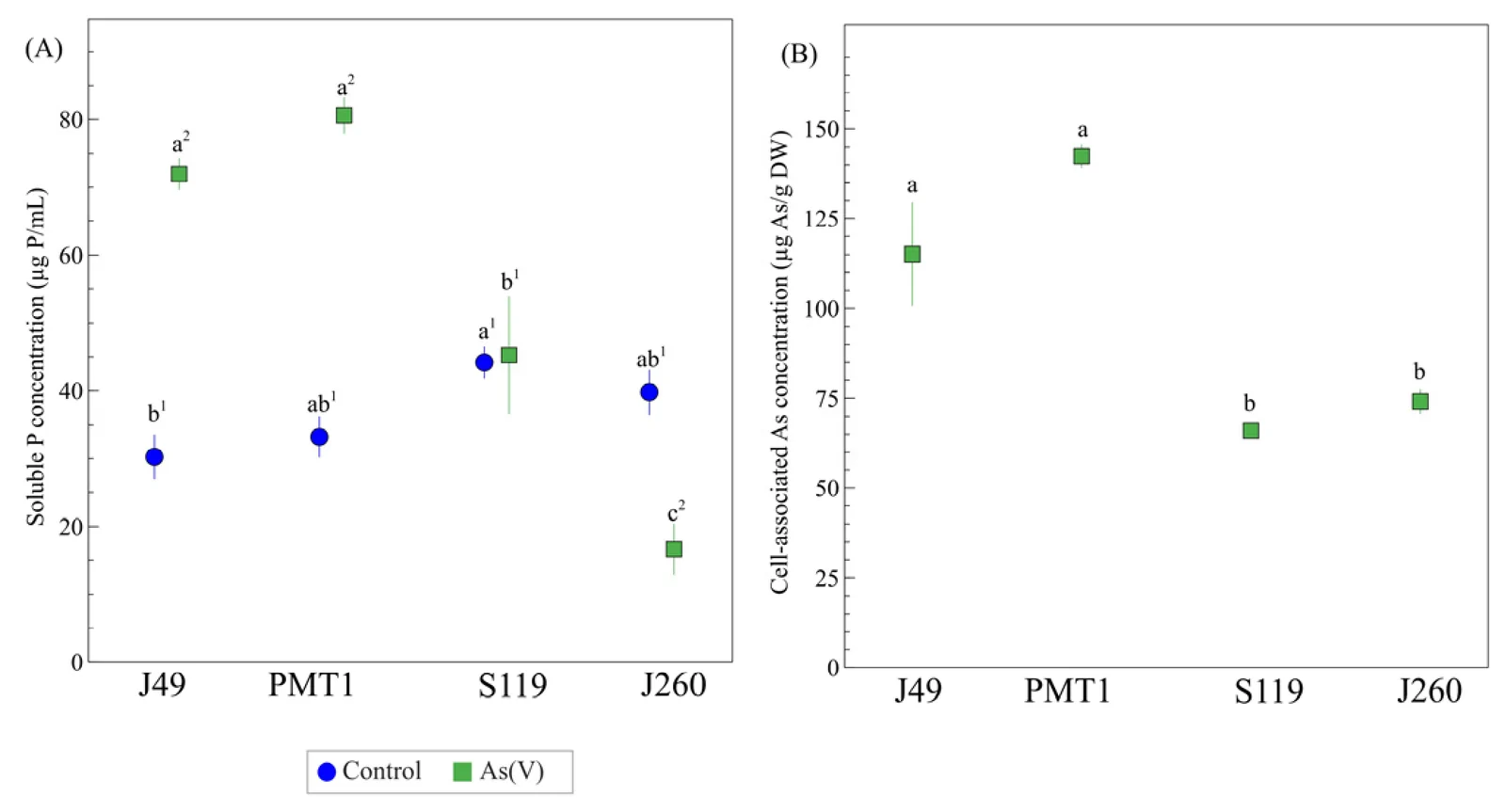
Phosphate solubilization and cell-associated metalloid concentration under strain-specific As(V) exposure. (**A**) Soluble P concentration in National Botanical Research Institute’s phosphate growth medium after 12 h of incubation. (**B**) Cell-associated As after exposure to the selected strain-specific As(V) concentrations. Blue symbols represent cultures grown without added As(V) (Control), whereas green symbols represent cultures exposed to the corresponding strain-specific As(V) concentration. J49 and PMT1 were exposed to 4 mM As(V), whereas S119 and J260 were exposed to 50 µM As(V); cultures grown without As(V) were used as controls. Values are presented as means ± SE. Exact sample sizes for each treatment × As(V) × variable combination are provided in Table S2. In panel (**A**), different lowercase letters indicate significant differences among strains within the same exposure condition, whereas different superscript numbers indicate significant differences between Control and As(V) within the same strain, according to Tukey’s test (*p* < 0.05). In panel (**B**), different lowercase letters indicate significant differences among strains under their respective As(V) exposure conditions according to Tukey’s test (*p* < 0.05). Because strain-specific As(V) concentrations were used, comparisons among exposed strains describe the conditions tested and should not be interpreted as equal-dose comparisons.

**Figure 3:**
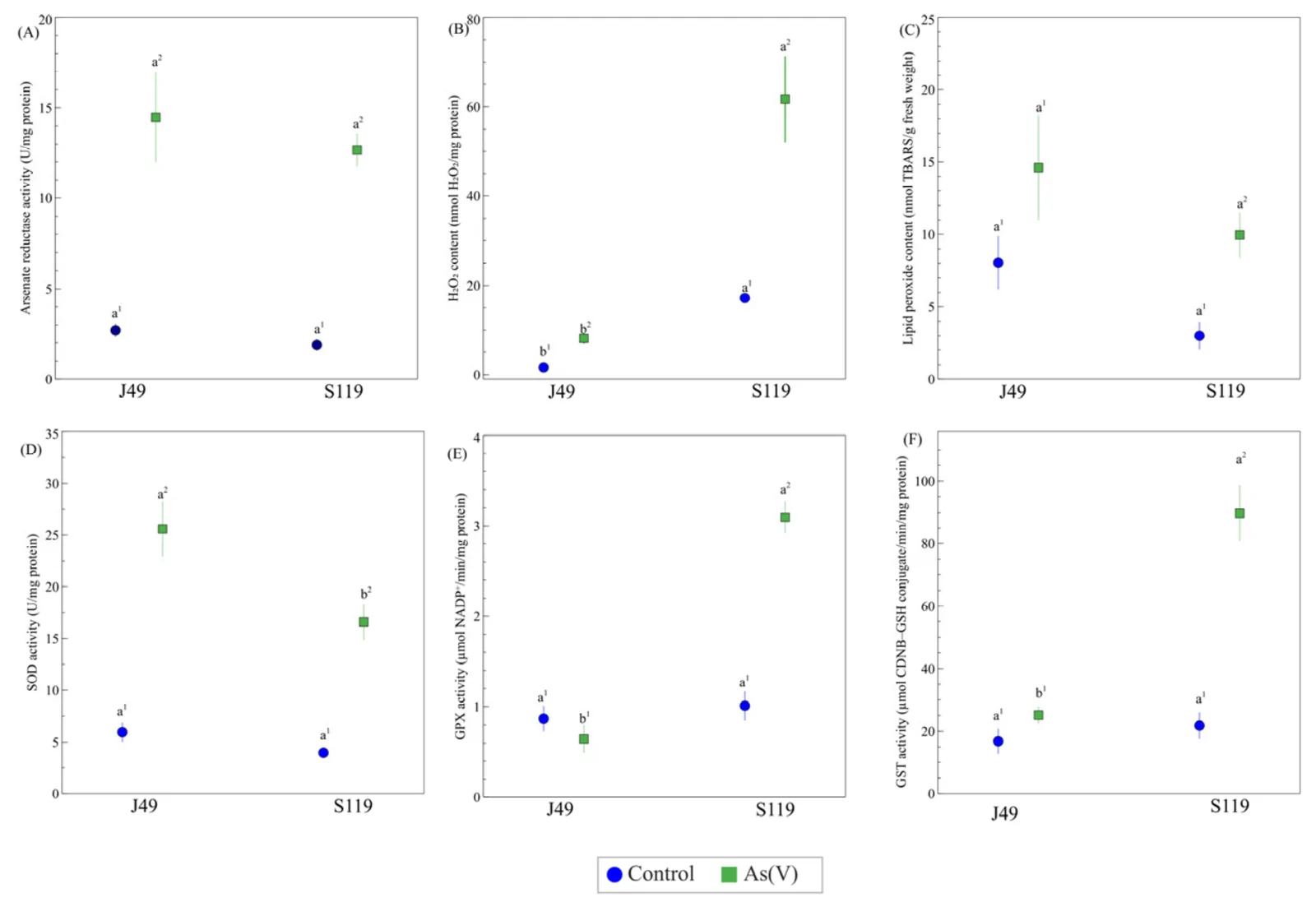
Biochemical responses of J49 and S119 to strain-specific As(V) exposure. (**A**) Arsenate reductase activity, (**B**) H_2_O_2_ content, (**C**) lipid peroxidation expressed as TBARS content, (**D**) superoxide dismutase (SOD) activity, (**E**) glutathione peroxidase (GPX) activity, and (**F**) glutathione S-transferase (GST) activity. J49 and S119 were exposed to 4 mM and 50 µM As(V), respectively; cultures grown without As(V) were used as controls. Values are presented as untransformed means ± SE (n = 3–11 independent biological replicates, depending on the strain, condition and assay). Within each exposure condition, different lowercase letters indicate significant differences between strains, whereas different superscript numbers indicate significant differences between Control and As(V) within the same strain (*p* < 0.05). Data were transformed when necessary to meet the assumptions of the statistical analyses. Because strain-specific As(V) concentrations were used, comparisons between As(V)-exposed strains represent values under their respective exposure conditions and should not be interpreted as equal-dose comparisons.

**Figure 4:**
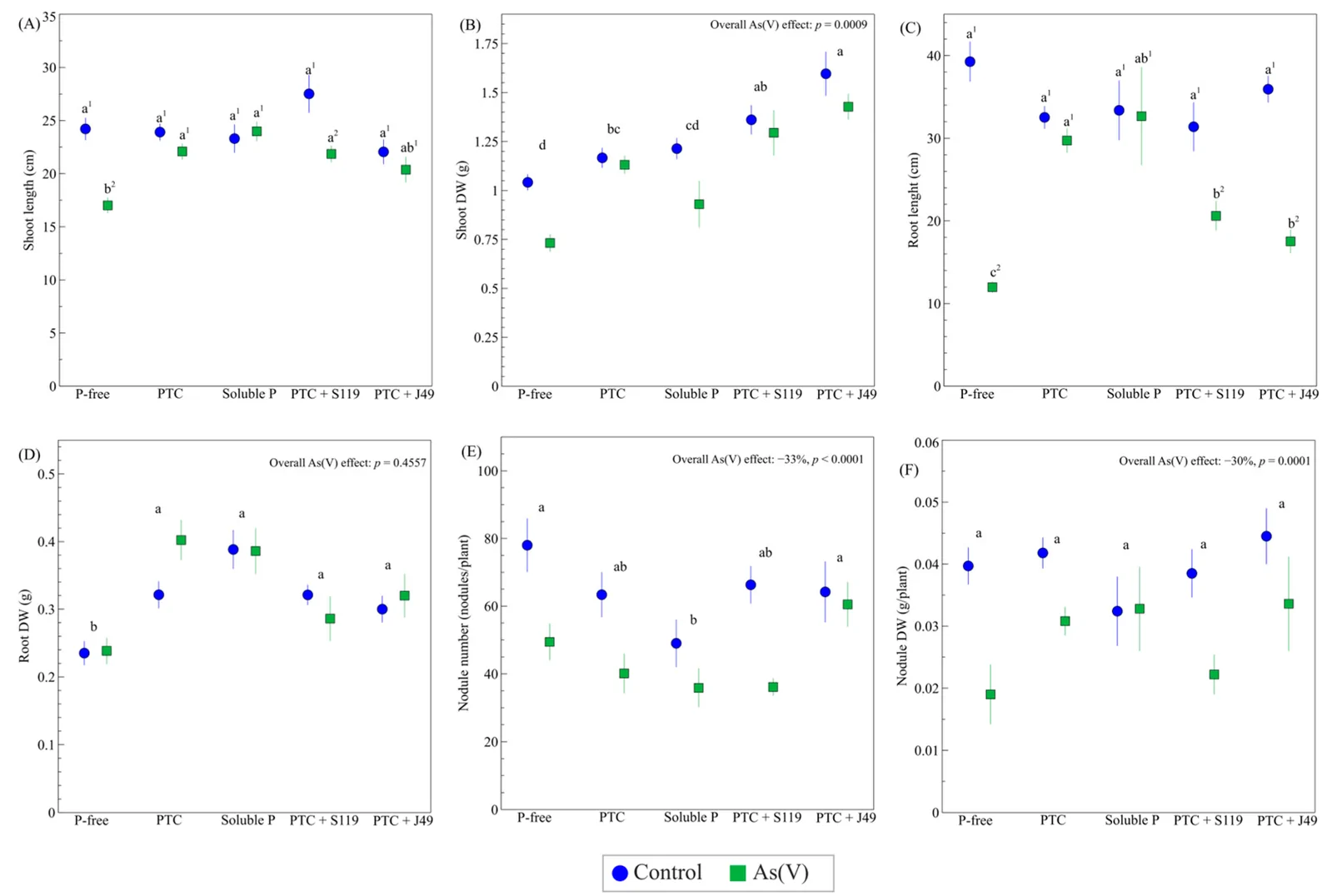
Effects of P availability, PS-PGPB coinoculation, and As(V) exposure on peanut growth and nodulation. (**A**) Shoot length, (**B**) shoot dry weight, (**C**) root length, (**D**) root dry weight, (**E**) nodule number, and (**F**) nodule dry weight. Blue circles represent plants grown without added As(V) (Control), whereas green squares represent plants exposed to 3 µM As(V). Plants were grown under five conditions: P-free, PTC, soluble P, PTC + S119, and PTC + J49, either without As(V) (Control) or with As(V). Values are presented as untransformed means ± SE. Sample sizes ranged from three to fourteen independent biological replicates, depending on the panel, treatment and As(V) condition. Exact sample sizes for each treatment × As(V) × variable combination are provided in Table S2. For panels A and C, in which the Treatment × As(V) interaction was significant, different lowercase letters indicate significant differences among growth conditions within the same As(V) condition, whereas different superscript numbers indicate significant differences between Control and As(V) within the same growth condition (*p* < 0.05). Shoot-length comparisons were performed on normal-score-transformed data using Tukey’s test; root-length comparisons were based on heteroscedasticity-robust analyses with Holm-adjusted *p* values.

**Table 1:** Plant growth-promoting traits of bacterial strains under strain-specific As(V) exposure.

Strain	As(V) Concentration	Potential Diazotrophic Growth		Siderophore Production		Phosphate-Solubilization Halo (cm)	
		Control	As(V)	Control	As(V)	Control	As(V)
J49	4 mM	+	+	+	−	1.30 ± 0.07 a ^1^	0.91 ± 0.07 b ^2^
PMT1	4 mM	+	+	+	−	1.37 ± 0.05 a ^1^	0.72 ± 0.12 bc ^2^
S119	50 µM	+	+	−	−	0.83 ± 0.03 b ^1^	1.21 ± 0.06 a ^2^
J260	50 µM	+	+	+	−	0.78 ± 0.07 b ^1^	0.51 ± 0.03 c ^2^

Values for the phosphate-solubilization halo are means ± SE. Different lowercase letters indicate significant differences among strains within the same As(V) condition, whereas different superscript numbers indicate significant differences between the control and As(V) conditions within each strain, according to Tukey’s test (*p* < 0.05). The symbols “+” and “−” indicate detection and no detection of the corresponding plant growth-promoting trait, respectively. Two-way analysis using HC3 heteroscedasticity-consistent standard errors showed significant effects of strain (F_3_,_56_ = 23.27, *p* < 0.0001), As(V) exposure (F_1_,_56_ = 21.52, *p* < 0.0001), and the strain × As(V) interaction (F_3_,_56_ = 23.85, *p* < 0.0001).

**Table 2:** Shoot N content and P concentration in peanut plants under control and As(V) conditions and different P and PGPB treatments.

Growth Conditions	N Content (mg/Plant)		P Concentration (mg/g DW)	
	Control	As(V)	Control	As(V)
P-free	2.947 ± 0.041 c ^1^	2.067 ± 0.019 d ^2^	1.73 ± 0.25 c ^1^	1.75 ± 0.11 b ^1^
PTC	2.645 ± 0.034 d ^1^	3.175 ± 0.040 a ^2^	2.86 ± 0.11 ab ^1^	1.78 ± 0.12 b ^2^
Soluble P	3.172 ± 0.034 b ^1^	2.450 ± 0.015 c ^2^	2.16 ± 0.11 bc ^1^	2.98 ± 0.20 a ^2^
PTC + S119	3.143 ± 0.034 b ^1^	2.015 ± 0.008 d ^2^	3.31 ± 0.09 a ^1^	2.84 ± 0.16 a ^1^
PTC + J49	3.717 ± 0.019 a ^1^	2.682 ± 0.051 b ^2^	3.06 ± 0.45 ab ^1^	2.93 ± 0.20 a ^1^

Values are means ± SE. Exact sample sizes for each treatment × As(V) × variable combination are provided in Table S2. For each variable, different lowercase letters indicate significant differences among growth conditions within the same exposure condition, whereas different superscript numbers indicate significant differences between control and As(V) within the same growth condition, according to Tukey’s test (*p* < 0.05). P concentration was square-root transformed before analysis; untransformed means ± SE are presented.

**Table 3:** Arsenic concentrations in peanut organs under As(V) exposure and different P and PGPB treatments.

	µg As/g DW			
Growth Conditions	Root	Shoot	Nodule	TF
P-free	58.57 ± 5.95 ^a^	16.39 ± 4.55 ^a^	ND	0.28 ^a^
PTC	42.06 ± 9.39 ^a^	8.41 ± 0.93 ^a^	73.62 ± 2.43 ^a^	0.22 ^a^
Soluble P	39.69 ± 9.86 ^a^	6.88 ± 1.21 ^a^	15.59 ± 3.07 ^c^	0.22 ^a^
PTC + S119	54.62 ± 13.78 ^a^	ND	ND	**—**
PTC + J49	61.24 ± 7.00 ^a^	ND	57.43 ± 2.67 ^b^	**—**

Values are means ± SE. Exact sample sizes for each treatment and organ are provided in Table S2. Different lowercase letters within a column indicate significant differences among growth conditions according to Tukey’s test (*p* < 0.05). Statistical comparisons were performed only among treatments with quantifiable As concentrations. Treatments in which organ As remained below the method detection limit were reported descriptively as ND, were not assigned numerical surrogate values, and were not included in inferential comparisons. Accordingly, lowercase letters apply only to treatments with quantifiable As concentrations. TF was calculated for each biological replicate as the ratio between shoot and root As concentrations. ND, As concentration below the method detection limit of approximately 1.5 µg As/g DW; —, not determined.

## Data Availability

The data presented in this study are available within the article and its accompanying Supplementary Materials.

## Supplementary Materials

The following supporting information can be downloaded at: https://www.mdpi.com/article/10.3390/plants15213332/s1. Figure S1: Growth curves of bacterial strains exposed to increasing As(V) concentrations over 12 h. (A) S119 and (B) J260 were exposed to 0–50 μM As(V), whereas (C) PMT1 and (D) J49 were exposed to 0–4 mM As(V). Viable cell counts were determined at 2-h intervals and expressed as log_10_(CFU/mL). Values are presented as means ± SE (n = 3 biological replicates). Table S1: Bacterial growth under increasing As(V) concentrations. Table S2: Exact number of independent biological replicates used for each variable, treatment, and As(V) condition; Data S1: Raw data and complete statistical outputs for all bacterial and plant variables.
