# Supplementary material for: Phosphate-Solubilizing Bacteria with Contrasting Arsenate Tolerance Sustain Phosphorus Nutrition and Restrict Arsenic Translocation in Peanut
Source: Plants (Basel). 2026 Oct 28;15(21):3332. doi: 10.3390/plants15213332 (PMC13766117; doi:10.3390/plants15213332)
Supplement: Supplementary file 1 [file plants-15-03332-s001.zip › Supplementary Table S1.pdf]

Supplementary Table S1. Bacterial growth under increasing As(V) concentrations

S119. log_{10}(CFU/mL)

Time (h)	0 µM	10 µM	20 µM	30 µM	40 µM	50 µM
2	7.90 ± 0.04 ab	8.03 ± 0.03 a	7.91 ± 0.01 ab	7.90 ± 0.04 ab	7.88 ± 0.03 b	7.89 ± 0.02 ab
4	9.04 ± 0.05 a	8.94 ± 0.08 ab	8.82 ± 0.02 abc	8.75 ± 0.06 bc	8.59 ± 0.06 cd	8.49 ± 0.03 d
6	9.87 ± 0.03 a	9.70 ± 0.06 a	9.25 ± 0.04 b	9.06 ± 0.03 c	9.13 ± 0.03 bc	8.85 ± 0.02 d
8	9.98 ± 0.06 a	9.90 ± 0.04 a	9.60 ± 0.09 b	9.42 ± 0.01 b	9.12 ± 0.07 c	9.09 ± 0.02 c
10	10.04 ± 0.02 a	9.96 ± 0.03 a	9.87 ± 0.02 ab	9.69 ± 0.08 b	9.16 ± 0.08 c	9.30 ± 0.00 c
12	10.08 ± 0.02 a	10.07 ± 0.04 a	10.07 ± 0.04 a	9.91 ± 0.02 a	9.69 ± 0.06 b	9.59 ± 0.04 b

J260. log_{10}(CFU/mL)

Time (h)	0 µM	10 µM	20 µM	30 µM	40 µM	50 µM
2	8.02 ± 0.02 a	8.08 ± 0.03 a	8.11 ± 0.10 a	8.11 ± 0.07 a	8.02 ± 0.04 a	8.04 ± 0.02 a
4	9.11 ± 0.01 a	8.95 ± 0.01 ab	8.76 ± 0.09 b	8.81 ± 0.03 b	8.80 ± 0.03 b	8.82 ± 0.03 b
6	9.83 ± 0.02 a	9.71 ± 0.04 a	9.40 ± 0.07 b	9.37 ± 0.04 b	9.31 ± 0.04 bc	9.14 ± 0.02 c
8	9.94 ± 0.04 a	9.85 ± 0.03 ab	9.78 ± 0.04 ab	9.71 ± 0.05 b	9.32 ± 0.03 c	9.25 ± 0.02 c
10	9.98 ± 0.03 a	9.85 ± 0.04 a	10.07 ± 0.12 a	9.80 ± 0.03 a	9.46 ± 0.04 b	9.33 ± 0.03 b
12	10.14 ± 0.01 a	9.93 ± 0.02 b	10.19 ± 0.07 ab	9.84 ± 0.04 ab	9.77 ± 0.09 abc	9.46 ± 0.03 c

PMT1. log_{10}(CFU/mL)

Time (h)	0 mM	1 mM	2 mM	3 mM	4 mM
2	7.94 ± 0.02 a	7.83 ± 0.06 ab	7.83 ± 0.06 ab	7.53 ± 0.12 b	7.65 ± 0.03 ab
4	8.96 ± 0.03 a	8.67 ± 0.05 b	8.54 ± 0.04 b	8.24 ± 0.04 c	8.10 ± 0.01 c
6	9.62 ± 0.02 a	9.25 ± 0.02 b	8.99 ± 0.05 c	8.87 ± 0.05 cd	8.72 ± 0.01 d
8	9.84 ± 0.05 a	9.75 ± 0.06 a	9.34 ± 0.10 b	9.26 ± 0.08 b	9.13 ± 0.06 b
10	9.92 ± 0.01 a	9.72 ± 0.04 b	9.58 ± 0.01 c	9.37 ± 0.05 d	9.19 ± 0.02 e
12	10.02 ± 0.04 a	9.72 ± 0.01 b	9.71 ± 0.03 b	9.44 ± 0.07 c	9.21 ± 0.02 d

J49. log_{10}(CFU/mL)

Time (h)	0 mM	1 mM	2 mM	3 mM	4 mM
2	7.86 ± 0.02 a	7.59 ± 0.05 ab	7.67 ± 0.15 ab	7.35 ± 0.04 b	7.41 ± 0.02 b
4	8.80 ± 0.10 a	8.50 ± 0.05 b	8.15 ± 0.05 c	7.84 ± 0.05 d	7.81 ± 0.02 d
6	9.58 ± 0.04 a	9.30 ± 0.16 a	8.61 ± 0.01 b	8.43 ± 0.01 b	7.70 ± 0.06 c
8	9.72 ± 0.03 a	9.28 ± 0.03 b	9.06 ± 0.02 bc	8.82 ± 0.06 cd	8.72 ± 0.09 d
10	9.83 ± 0.02 a	9.41 ± 0.05 b	9.16 ± 0.02 c	8.86 ± 0.05 d	8.57 ± 0.07 e
12	10.05 ± 0.03 a	9.61 ± 0.02 b	9.19 ± 0.03 c	9.14 ± 0.05 c	8.83 ± 0.06 d

Values are means ± SE (n = 3 biological replicates) and are expressed as log_{10}(CFU/mL). Different lowercase letters within the same strain and sampling time indicate significant differences among As(V) concentrations ($P < 0.05$). Following a significant As(V) concentration × time interaction in the repeated-measures model, concentration effects were analysed separately at each sampling time. Mean comparisons were performed using Tukey's test, except for J260 at 12 h, which was analysed using Welch's heteroscedastic one-way ANOVA followed by pairwise Welch comparisons with Holm-adjusted P values.
