# Supplementary material for: Phosphate-Solubilizing Bacteria with Contrasting Arsenate Tolerance Sustain Phosphorus Nutrition and Restrict Arsenic Translocation in Peanut
Source: Plants (Basel). 2026 Oct 28;15(21):3332. doi: 10.3390/plants15213332 (PMC13766117; doi:10.3390/plants15213332)
Supplement: Supplementary file 1 [file plants-15-03332-s001.zip › Supplementary Table S2.pdf]

TABLE S2. Exact number of independent biological replicates used for each variable, treatment, and As(V) condition.

VARIABLE	P-free Control	P-free As(V)	PTC Control	PTC As(V)	Soluble P Control	Soluble P As(V)	PTC + S119 Control	PTC + S119 As(V)	PTC + J49 Control	PTC + J49 As(V)
**Shoot length**	9	8	12	14	9	6	14	14	10	8
**Root length**	6	8	9	8	4	3	7	10	7	8
**Shoot DW**	7	6	6	7	8	4	6	6	6	4
**Root DW**	6	6	8	9	6	5	9	10	8	5
**Nodule number**	6	5	8	8	4	7	10	9	5	6
**Nodule DW**	6	4	4	9	5	4	11	6	6	5
**N content**	3	3	3	3	3	3	3	3	3	3
**P concentration**	3	3	3	3	3	3	3	3	3	4
**Root As**	—	3	—	3	—	3	—	3	—	3
**Shoot As**	—	3	—	3	—	3	—	3	—	3
**Nodule As**	—	3	—	3	—	4	—	3	—	3

DW, dry weight; —, not analyzed. Differences in sample size reflect the variable-specific sampling and material requirements. Morphological variables were measured in larger sets of individual plants, whereas destructive nutrient and organ-specific As determinations were conducted on analytical subsets. For nodule As determination, tissue from multiple plants belonging to the same biological replicate was pooled when necessary to obtain the required analytical mass.
